# A Pattern to Link Adenosine Signaling, Circadian System, and Potential Final Common Pathway in the Pathogenesis of Major Depressive Disorder

**DOI:** 10.1007/s12035-022-03001-3

**Published:** 2022-08-23

**Authors:** Xin-Ling Wang, Wilf Gardner, Shu-Yan Yu, Tsvetan Serchov

**Affiliations:** 1 School of Basic Medical Sciences, Cheeloo College of Medicine, Shandong University, Ji’nan, 250012 Shandong China; 2 Shandong Key Laboratory of Mental Disorders, School of Basic Medical Sciences, Cheeloo College of Medicine, Shandong University, Ji’nan, 250012 Shandong China; 3 Centre National de La Recherche Scientifique, Université de Strasbourg, Institut Des Neurosciences Cellulaires Et Intégratives, 67000 Strasbourg, France; 4 Department of Psychiatry and Psychotherapy, Medical Center - University of Freiburg, Faculty of Medicine, University of Freiburg, Freiburg, Germany; 5 University of Strasbourg Institute for Advanced Study (USIAS), 67000 Strasbourg, France

**Keywords:** Adenosine receptors, Circadian genes, Depression, *Homer1a*, CREB

## Abstract

Several studies have reported separate roles of adenosine receptors and circadian clockwork in major depressive disorder. While less evidence exists for regulation of the circadian clock by adenosine signaling, a small number of studies have linked the adenosinergic system, the molecular circadian clock, and mood regulation. In this article, we review relevant advances and propose that adenosine receptor signaling, including canonical and other alternative downstream cellular pathways, regulates circadian gene expression, which in turn may underlie the pathogenesis of mood disorders. Moreover, we summarize the convergent point of these signaling pathways and put forward a pattern by which *Homer1a* expression, regulated by both cAMP-response element binding protein (CREB) and circadian clock genes, may be the final common pathogenetic mechanism in depression.

## Introduction

Major depressive disorder (MDD) is one of the most prevalent forms of mental illness. It is a complex and heterogeneous disorder associated with high individual suffering, increased risk of suicide, and a severe economic burden for society [1]. Several lines of evidence from animal and human studies have shown that disturbances of circadian clockwork are associated with the development of depression. Moreover, different chronotherapies, a variety of strategies that modulate biological clock, such as sleep deprivation and light therapy, are considered as alternative treatments for depression [2]. However, how the circadian clock influences pathophysiology of mood disorders, as well as the molecular and cellular mechanisms of action of the therapeutic interventions targeting circadian rhythm, is not well understood. The identification of the neurobiological substrates mediating the crosstalk between the circadian clock and mood regulation may lead to the development of new strategies for prevention and treatment of depression.

Numerous studies have demonstrated a role of adenosine receptors in the development of depression and antidepressant therapies [3–5]. Moreover, adenosinergic signaling is implicated in the regulation of different aspects of the circadian clock [6, 7]. However, the detailed mechanism has not been completely clarified. Recently, we found that the canonical circadian clock genes *Per1* and *Per2* were involved in the antidepressant action of an adenosine A_1_ receptor (A_1_R) agonist [8]. In addition, it has been shown that the expression of the synaptic plasticity protein Homer1a, proposed by our group as an important element mediating antidepressant effects and also a downstream target of adenosine receptor signaling [9–12], is directly regulated by the circadian clock [13].

In the present article, we review relevant recent advances linking adenosine receptors, circadian clock, and mood and propose that adenosine signaling regulates circadian clockwork and Homer1a, which may be a potential final common mechanism involved in the neurobiology and treatment of depression.

## Adenosine Signaling and Mood

There are numerous studies on adenosine signaling and depression, which have been recently reviewed extensively by others [3–5]. The cellular effects of adenosine are mediated by four subtypes of G-protein coupled receptors: A_1_R, A_2A_R, A_2B_R, and A_3_R. In general, it was proposed that A_1_Rs promote antidepressant-like effects, while A_2A_Rs’ activation enhances depression-like behaviors in rodents [3]. As for the A_2B_ and A_3_ receptors, at present, we could not find any reports on their role in mood disorders [14, 15].

Several non-pharmacological antidepressant treatments including sleep deprivation (SD), electroconvulsive therapy (ECT), and deep brain stimulation (DBS) enhance A_1_R signaling [9]. Hines et al. was first to demonstrate that A_1_Rs are necessary for the antidepressant action of SD and that their activation leads to rapid antidepressant-like effects [16]. Our group utilized a line of transgenic mice conditionally overexpressing A_1_R in calcium/calmodulin-dependent protein kinase type II (CaMKII) forebrain neurons [9, 11, 17]. Upregulating A_1_R led to pronounced acute and chronic resilience toward depressive-like behavior in various tests, while A_1_R knockout mice displayed an increased depressive-like behavior and were resistant to the antidepressant effects of SD [9]. Furthermore, we have shown that the antidepressant effects of A_1_R activation are mediated by the synaptic plasticity protein Homer1a, which is upregulated by various antidepressant treatments such as SD, imipramine, ketamine, and A_1_R activation [9, 12]. Using a different transgenic mouse lines with overexpression of A_1_R in the cortex and hippocampus, we found that depending on the brain region of A_1_R upregulation, the mice show different resistance to depression-like behavior, and that enhanced Homer1a expression in the hippocampus increases stress vulnerability [11].

However, activation of A_1_R may elicit also manic or hypomanic episodes in patients with bipolar disorder [18]. It has been reported that peripheral adenosine levels were negatively correlated to the severity of depressive symptoms of bipolar disorder patients [19]. Therefore, peripheral adenosine levels may have a positive relationship with mood, demonstrating the pivotal role of adenosine in mood regulation.

In contrast, it has been reported that rats with A_2A_R overexpression in hippocampus, cortex, and striatum show increased depression-like behavior [20]. Vice versa, A_2A_R KO mice exhibit reduced depression-like behaviors, such as decreases in the immobility time in forced swimming test and tail suspension test [21, 22]. The A_2A_R antagonist istradefylline (KW6002) showed an antidepressant-like action on learned helplessness model rats [23]. However, some contradictory results of relationship between A_2A_R and mood have also been released. Tsai et al. reported that they did not find any association of A_2A_R (1976C > T) genetic polymorphism with mood disorders [24]. However, this does preclude the possibility of a role of A_2A_R in the pathogenesis of mood disorders; rather, other A_2A_R variants must also be extensively studied. Moreover, A_2A_Rs have also been linked with depression, suicidal behavior, and impulsivity based on indirect evidence at a statistical association level [25]. For instance, Lucas et al. reported a negative association between caffeine consumption and risk of suicide based on cohort studies [26]. The actions of adenosine receptors on depression are summarized below (Table 1).

**Table 1:** Effects of adenosine receptors on depression or depression-like behavior

Receptors	Effect of activation	References
A_1_R	Antidepressant like effect	[3, 8, 9, 11, 16, 139]
A_2A_R	Pro-depressive like effect	[3, 20–23, 25, 26, 139, 140]

## Circadian Clock and Mood Regulation

Circadian clocks govern a wide range of biochemical, physiological, and behavioral processes. In mammals, the circadian master pacemaker is located in the suprachiasmatic nucleus (SCN) [27]. The circadian oscillation of the intracellular clock is driven by transcription/translation-based feedback/feedforward loops, consisting of a set of clock genes. Positive regulatory elements are brain and muscle ARNT-like 1 (BMAL1) and circadian locomotor output cycles kaput (CLOCK), which form heterodimers and induce the rhythmic transcription of *Period* (*Per1* & *Per2*) and *Cryptochrome* (*Cry1* & *Cry2*) genes. The PER and CRY proteins interact and translocate to the nucleus, where they act as negative regulators inhibiting CLOCK/BMAL1 transcription [28]. An additional loop including both activating and repressing regulatory elements is formed by retinoic acid receptor-related orphan receptors (ROR α, β, and γ) and nuclear receptors REV-ERB (α & β) [29, 30].

A large number of studies have demonstrated the relationship between the circadian clock and depression [31–39], with a great many reviews to refer to [31, 32, 34, 36, 40–45]. Most of these reports have shown correlation between genes, RNAs, proteins, and single nucleotide polymorphisms with the symptoms of MDD or depression-like behaviors [33, 38, 39, 46–48]. For example, variants of circadian genes, such as *CLOCK*, *BMAL1*, *NPAS2*, *Per3*, and *NR1D1*, play a role in mood disorders, mainly based on statistical analyses [49–53]. In addition, transgenic mice with mutations in certain clock genes have been characterized with depressive-like behavior. However, each mouse model shows a distinct mood/rhythm combination phenotype: similar mood characteristics occur with opposite changes of circadian period, and reduced circadian amplitude leads to different changes in mood behavior, which hinders a clear conclusion/hypothesis [54].

Several brain regions relevant to psychopathology of depression, including the prefrontal cortex, hippocampus, amygdala, lateral habenula (LHb), and nucleus accumbens (NAc), possess an oscillating molecular clock [55–57]. Increasing evidence from human and rodents suggests that these region-specific oscillators in limbic areas are instrumental regulators of mood. Indeed, a microarray study demonstrated that the circadian patterns of gene expression in six brain regions (including amygdala, prefrontal cortex, hippocampus, and NAc) are significantly altered in human postmortem subjects with MDD [48]. Moreover, many chronic stress-based animal models of depression show dysregulated circadian rhythms of locomotor activity, body temperature, and corticosterone levels [58], as well as reduced circadian expression amplitude of several canonical circadian clock genes in the SCN and amygdala, but increased amplitude in the NAc [55, 56, 59]. For instance, Christiansen et al. demonstrate effects of chronic mild stress on core circadian genes in rats [46]—the mean peak times of *Per2* and *Bmal1* expressions in SCN were either phase-delayed or phase-advanced in the chronic stress group. Taken together, these reports suggest that stress and/or MDD might differently affect the circadian clockwork in particular brain areas and that further investigation on region-specific circadian mechanisms are needed.

A potential role has been recently proposed for the circadian clock in the mechanism of rapid antidepressant treatments, like SD and ketamine [60]. Duncan et al. revealed an association between ketamine’s clinical antidepressant response and circadian-related wrist-activity parameters [39], finding that responders showed a phase-advanced activity rhythm and a decreased measure compared with nonresponders at baseline. Orozco-Solis et al. showed downregulation of several canonical clock genes, including *Per1*, *Per2* and *Cry2*, by rapid antidepressant therapies SD and low-dose ketamine, using comparative transcriptomics analyses [38]. Furthermore, ketamine usually takes its most robust effect on the next day of its treatment [61], a phenomenon probably related to the effect of ketamine on circadian system [43]. Preclinical studies reveal that both SD and ketamine downregulate circadian genes, probably through NMDAR, AMPAR, TrkB, MAPK, mTOR, GSK3β, and CREB [38, 62–65], but the exact cellular pathway has not been confirmed and needs to be further investigated. Until now, only a few studies have revealed signaling pathways that act directly on the molecular circadian clock and mediate the pathogenesis of major depression or depression-like behaviors [8, 66].

The role of circadian rhythm in mood regulation is bidirectional, affecting both depression and mania [67–75]. For example, phase advance during manic episodes and phase delay during depressive episodes were found in the patients with bipolar disorder [76–79]. The *CLOCK*^Δ19^ and *Per2*^Brdm1^ mice exhibit hyperdopaminergic state and mania-like phenotypes [80–82], while in contrast, *Per1* knockout mice show depression-like behavior in forced swim test [83], directly demonstrating that the circadian clock influences monoamine oxidase A and mood. In addition, Olejniczak et al. revealed that light affects depression-like behavior through *Per1* in the LHb [83]. Therefore, our focus should not be restricted to only one axis of investigation. For instance, while A_1_R agonism shows antidepressant-like effects, it may potentially induce manic or hypomanic episodes and vice versa [18, 84]. As a result, this side effect must be avoided when exploring novel antidepressants or mood stabilizers. Recently, Hinton et al. reported that administration of caffeine during adolescence in mice could induce circadian-dependent changes in mood fluctuations in adulthood, including depression and mania [85]. However, the exact cellular pathway underlying this phenomenon needs to be investigated further. In future, elucidation of the pathogenesis of trans-phase may be an important research field.

Taken together, these reports support a causal relationship between the circadian system and mood. However, alternative hypotheses have been proposed, and whether the disruption of circadian clocks are causes or consequences of mood disorders remains undetermined. Accordingly, Lazzerini Ospri et al. provide a model suggesting that mood may be an output of circadian rhythm by probability [86]. However, this hypothesis also needs to be further verified.

## The Role of Adenosine Receptors in Circadian Clock Modulation

Light is the most potent resetting stimuli of the circadian clock. In addition to glutamate, adenosine appears to be a strong candidate for modulating SCN activity [87]. Indeed, application of adenosine attenuates light-induced phase shifts, while A_1_R antagonism can reverse this effect [88, 89]. Adenosine is known to increase during SD [90] and accordingly it has been shown in rodents and humans that SD also reduces the photic resetting of circadian activity [91, 92]. Likewise, in response to acute SD, a subset of circadian clock genes behave as immediate early genes and are transcriptionally responsive within hours of treatment [93, 94]. Conversely, longer SD suppresses 80% of rhythmic genes in the mouse brain [95, 96]. Moreover, the adenosine receptor antagonist caffeine modulates different aspects of the circadian rhythms including behavioral rhythm and the molecular clock [87, 97, 98]. It increases the light-entraining activity rhythm and lengthens the period of *hPer2* and *mBmal1* [97, 99]. In human-cultured cells, caffeine produced its effect on the circadian clock through adenosine receptor-cAMP signaling [100].

## Adenosine A_1_ R and A_2A_R Signaling Pathways as Regulators of the Molecular Circadian Clock and Mood

In the following chapter, we will review and discuss A_1_R and A_2A_R downstream signaling, including classical pathways and some alternative cascades, implicated in the regulation of the cellular circadian system and mood. Moreover, transcriptional factor CREB phosphorylation and induction of the synaptic protein Homer1a appear to be a convergent point of various pathways [101], and play a critical role both in the regulation of circadian rhythm [102, 103] and in the pathogenesis of depression [101].

### Canonical Adenosine Signaling

#### ERK MAPK Pathway

**Fig. 1:**
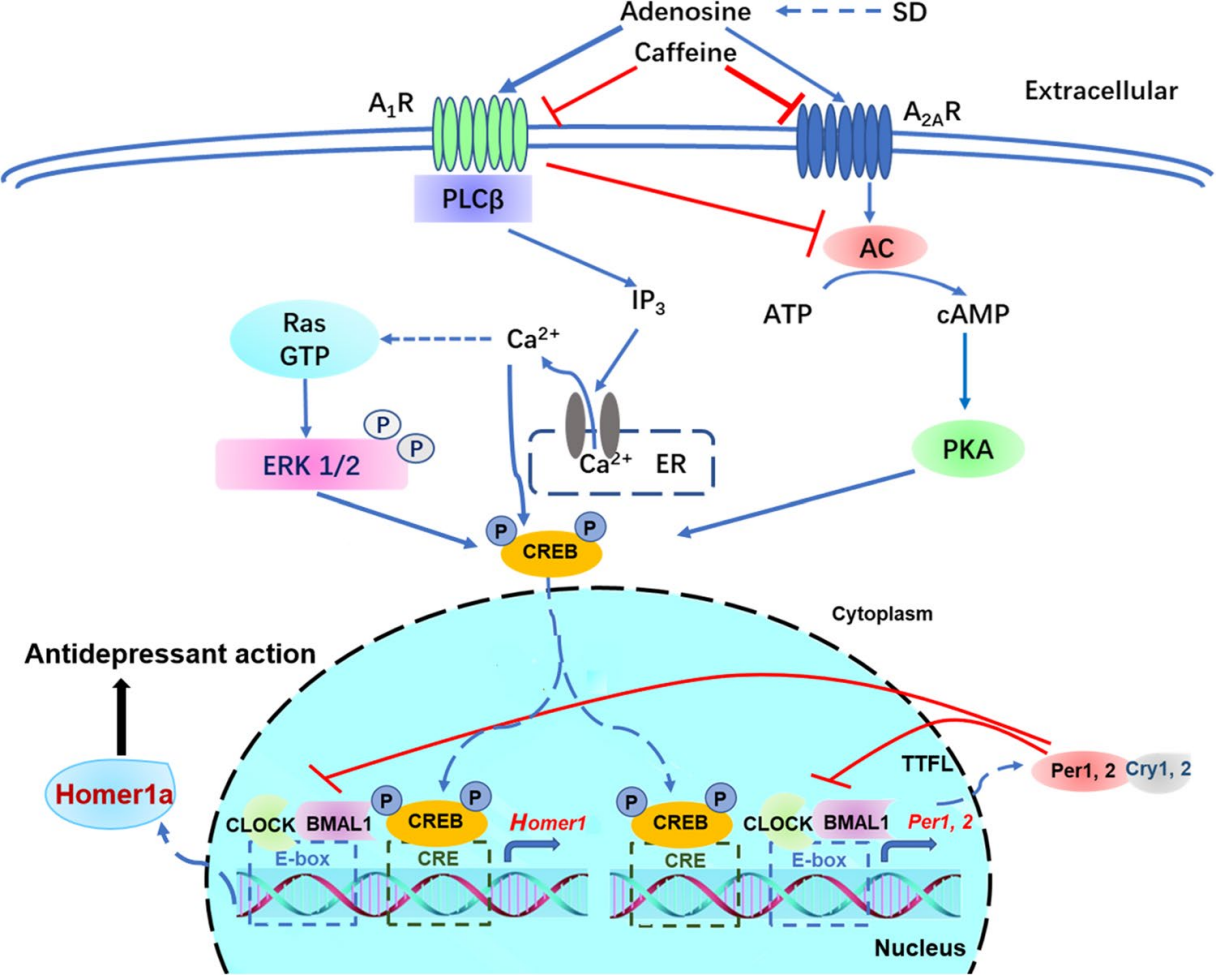
Pattern of a potential final common mechanism of antidepressant action. Acute or chronic SD increases the adenosine levels in the brain and activates A_1_Rs and A_2A_Rs. Subsequently, ARs act on various signaling pathways, including cAMP, PKA, Ca2 + , and MAPK in the cytoplasm, and converge on the phosphorylation of the transcriptional factor CREB. The phosphorylated CREB enters the nucleus and binds with the CRE sites on *Homer1* and *Per1/2* promoters to regulate their transcription. Simultaneously, these genes are transcriptionally regulated by CLOCK/BMAL1 via E-box elements on their promoters. Concurrently, PER form complexes with CRY in the cytoplasm, which in turn compete with CLOCK/BMAL1 complexes and block their transcription, as well as Homer1a expression. SD, sleep deprivation; AC, adenylate cyclase; PLCb, phospholipase C beta; ATP, adenosine triphosphate; cAMP, cyclic adenosine monophosphate; IP3, inositol triphosphate; PKA, protein kinase A; ER, endoplasmic reticulum. Arrows indicate activation: the thicker arrow indicates that adenosine may have a preferential activation effect on A_1_R during acute SD. In parallel, the red lines indicate inhibition, where the thicker line indicates greater inhibition of A_2A_R by caffeine

A_1_R activates the phospholipase C (PLC)β—inositol triphosphate (IP_3_) pathway in order to induce the release of calcium from endoplasmic reticulum and subsequently activates extracellular regulated protein kinase (ERK) [10, 104]. After ERK is activated, it can consequently activate the downstream part of the MAPK signaling pathway. CREB is the endpoint of the pathway, which can enter the nucleus and bind to the CRE sites in the promoter regions of *Homer1a*, *Per1*, and *Per2* genes to regulate their transcription (Fig. 1) [10, 38, 44, 103, 105–107]. Moreover, it has been reported that adenosine A_1_R-ERK1/2 signaling pathway in the prefrontal cortex and hippocampus region of mice was involved in the anti-menopausal depressant-like effect of Jiao-Tai-Wan [108]. Additionally, there have been numerous references showing that the ERK MAPK pathway plays a critical role in MDD [109–113]. Moreover, ERK-CREB signaling in the hippocampus and prefrontal cortex was revealed as the downstream pathway of inosine to produce its antidepressant-like effect [114]. Thus, the ERK MAPK pathway is both important in circadian systems and mood regulation.

#### cAMP Signaling Pathway

cAMP is also a classical downstream signaling pathway of both A_1_R and A_2A_R and plays a key role in the mammalian circadian clock [100, 105, 115]. The A_1_R can suppress the cAMP pathway through inhibiting adenylate cyclase (AC) via its G_i_; contrarily, the adenosine A_2A_ receptor can stimulate cAMP pathway through activating AC via its G_s_ (Fig. 1) [3]. Burke et al. found that the intracellular mechanism of caffeine-induced regulation of the circadian rhythm is via the adenosine A_1_ receptor-cAMP signaling pathway in human cells in vitro [100]. In addition, it was revealed that the cAMP-protein kinase A (PKA)-CREB pathway in rat hippocampal neurons was involved in the antidepressant-like effect of serum [116]. However, no interaction was identified of this pathway with circadian genes. As CREB is the endpoint of various cellular pathways, including the cAMP pathway, it has been suggested that CRE sites on the *Per1* or *Per2* genes might be the potential target (Fig. 1) [103, 105, 106]. Therefore, cAMP is another downstream signaling pathway that plays critical roles in both regulation of circadian genes and mood.

#### ***Ca***^***2***+^***Signaling Pathway***

Studies have revealed that both L-type calcium channels and calcium-induced calcium release can induce post-synaptic adenosine elevation [117] and that calcium signaling acts upon *Per1*/*2* genes directly via CREB in mammalian cells [103, 115, 118]. In addition, A_1_R can also inhibit L-type calcium channels via its G_i_ [3]. Besides this, the ERK/MAPK signaling pathway is calcium-dependent. It has also been reported that the G_q_-Ca^2+^ axis controls the circadian clock in the SCN [119], involved in both input and output of circadian systems [115, 120]. Furthermore, the cAMP/Ca^2+^ signaling pathway determines properties of the circadian system, including phase, amplitude, and period; in turn, cAMP/Ca^2+^ signaling is regulated by circadian system and rhythmically expressed [115].

In conclusion, ERK MAPK, cAMP, and Ca^2+^ signaling pathways are the major downstream pathways of adenosine, which in parallel can regulate circadian molecular clock (Fig. 1). Pertinently, it has been demonstrated that levels of cAMP, Ca^2+^, ERK, and CREB were decreased in postmortem patients with MDD [121]. Moreover, levels of these molecules were oppositely altered in patients with bipolar disorder treated with mood stabilizers compared to MDD patients administered antidepressants [121], demonstrating their roles in mood regulation.

### Other Potential Alternative Downstream Cellular Pathways of Adenosine Receptors

In addition to the canonical cellular pathways, there are also some recently explored downstream signaling pathways of A_1_R, which have not been demonstrated to be involved in mood regulation but may suggest new further research directions.

Recently, Jagannath et al. revealed that adenosine could regulate the circadian clock through activating the adenosine A_1_/A_2A_ receptor, and their downstream Ca^2+^-ERK-AP-1 and CREB/cAMP-regulated transcriptional coactivators (CRTC1)-CRE signaling pathways to modulate the expression of *Per1* and *Per2* genes in mice [122]. They found that these signaling pathways were also stimulated by light [122]. Thus, adenosine can alter the circadian time by integrating signals from light and sleep. Furthermore, Trautmann et al. showed that caffeine acts on mood through the elevation of phosphorylated Thr75-DARPP-32, which can bind to CLOCK and inhibit the CLOCK/BMAL1 complex interaction, consequently modulating the expression of circadian genes and potentially linking adenosine, circadian systems, and mood [66].

Adenosine receptors are also involved in the modulation of other neurotransmitter systems. For example, the A_2_AR is colocalized postsynaptically in dopamine areas, including the striatum and NAc [123]. Indeed, it has been demonstrated that there is a functional interaction between dopamine D2Rs and A2ARs, which converge on the same signal transduction pathways in an antagonistic way [124]. Likewise, A1R and D1Rs antagonistically interact [125]. The dopaminergic system plays an important role in the control of reward and motivation-oriented behavior, which is severely affected in MDD. Since dopamine synthesis and particularly its limiting enzyme tyrosine hydroxylase (TH) are under circadian regulation, this interaction between adenosinergic and dopaminergic system represents another potential signaling pathway involved in mood regulation [126].

### Convergent Points of Adenosine Receptor Signaling

#### CREB

CREB is a convergent point of various pathways in the pathogenesis of MDD and is the downstream effector molecule of adenosine signaling [101]. The role of CREB in MDD varies with different brain regions [101]. For example, overexpression of CREB in the dentate gyrus of the hippocampus produced an antidepressant-like effect in rats [127], while overexpression of CREB in either the CA1 pyramidal cell layer of the hippocampus or the prefrontal cortex did not show this effect [127]. Conversely, overexpression of CREB in the basolateral amygdala or in the NAc produced a pro-depressive-like effect [128, 129]. Meanwhile, the acting points of CREB on the circadian genes *Per1*/*2* have been elucidated (Fig. 1) [8, 106, 122]. Phosphorylated CREB is one of the transcriptional factors regulating *Per1*/*2*. Besides this, AP-1 is another transcriptional factor that can also bind with AP-1 sites in the promoter regions of *Per* genes. In the *Per2* gene, AP-1 REs are putative and conserved, while in contrast are not well conserved in *Per1* [122]. Moreover, it has been reported that the sequences of CRE (TGACGTCA) and 12–0-tetradecanoylphorbol-13-acetate-responsive element (TRE) (TGACTCA) are very similar, and that the nuclear factors of CREB, CRE modulator (CREM), and Jun were also very similar in structure [130, 131]. This may lead to transcriptional cross-talk and potential competitive effects. Therefore, we deduce that this physiological process may be involved in the interaction between *Per1*, *Per2*, and *Homer1a* genes, and may play a key role in supplement to the traditional feedback loops of the molecular circadian clock.

CREB conduction signals can also be regarded as an intrinsic part of clock oscillations, modulating acute alterations in the circadian clock and transcription-translation feedback loops [118, 132].

Apart from circadian genes *Per1*/*2*, there are hundreds of genes that have CRE sequences in their promoter regions that can be bound with pCREB/CREB. Therefore, *Per1*/*2* might not be the only final common targets of antidepressants and other genes, such as *Homer1a*, might have an interaction with these circadian genes (see below).

#### Homer1a

Homer1a is a member of the Homer family of postsynaptic scaffolding proteins, which is rhythmically expressed and acts as neuronal activity-inducible modulator of glutamatergic signaling [10, 13, 133]. It has been shown that Homer1a induction, as a downstream effect of A_1_R signaling, may be a convergent point of several non-pharmaceutical treatments of MDD [9–11, 133]. Homer1a has been subsequently proposed as a final common pathway of various antidepressant therapies, including ECT, TMS, SD, and ketamine, as well as for classical treatments, such as imipramine and fluoxetine [10]. In addition, it has been shown that metabotropic glutamate receptor 5 (mGlu5) and α-amino-3-hydroxy-5-methyl-4-isoxazole-propionicacid receptor (AMPAR) might be the potential targets for Homer1a to act and exhibit its antidepressant effect [12]. Recently, Sato et al. revealed that the *Homer1* gene is bimodally regulated by CREB via the CRE site and by the CLOCK/BMAL1 complex via E-box, demonstrating an important crosstalk between CREB and the circadian clock, and thus showing a pivotal role of Homer1a in integrating signals from both adenosine signaling and circadian rhythms [13]. Therefore, this may be the most promising final common pattern in the pathogenesis of depression and the mechanism of antidepressants. Thus, we propose that *Homer1* and *Per* genes, receiving signals from both CREB and the CLOCK/BMAL1 complex, which is inhibited by PERs, may be a potential common mechanism of various antidepressant therapies (Fig. 1).

## Conclusions and Future Directions

Acute SD is known to elicit rapid antidepressant effects, while chronic sleep restriction is considered as a risk factor for depression [134]. However, adenosine is accumulated in the brain after both acute and chronic sleep loss and acts as modulatory neurotransmitter regulating brain homeostasis via modulation of sleep and homeostatic plasticity, circadian clockwork, and mood [3, 9, 135–138].

We deduce that, on one hand, this conflicting effect of adenosine might be due to the preferential activation of its receptors, since A_1_R and A_2A_R signaling have contrasting effects on mood. Perhaps during the acute SD phase, adenosine has a greater effect on A1R [9], whereas, during chronic sleep loss, there may be a counterbalancing effect and possibly more action on the A_2A_R with an opposing effect on downstream signaling (Serchov et. 2020). At the same time, caffeine, an antagonist of adenosine receptors, may have a stronger antagonistic effect on A2AR than A1R, resulting in an antidepressant effect [21, 22, 139]. On the other hand, pCREB may also have a biased or counterbalancing effect on *Homer1* and *Per* genes, and the final Homer1a protein expression level may depend on the probability of circadian output, which may match the alternative hypothesis model provided by Lazzerini Ospri et al. [86].

As discussed above, circadian gene expression is differentially affected by chronic stress, depression, or antidepressant treatments in different brain regions. Thus, the different effects of adenosine signaling on the circadian clock, Homer1a expression, and mood might also be brain region-specific [11, 133].

Taken together, we summarize that adenosine A_1_R/A_2A_R signaling converges on the transcriptional factor CREB. After CREB is activated, it can bind to both CRE/AP-1 sites on *Per1*/*2* gene promoters, or the CRE site on the *Homer1* promoter, thus modulating their expression. In turn, Per/Cry complexes translocate to the nucleus and inhibit BMAL1 activity. Since Homer1 expression is bimodally regulated by BMAL1 and CREB, we deduce that Homer1a expression might be inhibited by Per indirectly (Fig. 1). Thus, Homer1a is potentially a final common pathway in the pathogenesis and treatment of depression, which links adenosine signaling, circadian clock, and neuro-plasticity together, mediating both the antidepressant effects of acute SD and the detrimental action on mood of chronic sleep loss.

Additionally, the synthesis of dopamine is also regulated in a circadian manner, through the time-dependent expression of TH by promoter occupancy of CLOCK, NAD + -dependent sirtuin 1 (SIRT1), and CREB [107]. This pathway links the metabolic system, circadian rhythms, and neurotransmitter system together, important in the regulation of many physiological processes and psychiatric diseases. In the future, we shall investigate this common mechanism from several other perspectives: neuro-inflammation, systems of monoamine and glutamatergic signaling, the hypothalamic–pituitary–adrenal (HPA) axis, brain-gut axis, metabolic peptide signal transduction, and mitochondrial function, with the aim of exploring the common pathophysiology of depression from a cellular to systemic level.

Above all, identification of the common pathogenesis of MDD will help us to better understand the underlying pathogenesis of mood disorders and to explore novel antidepressants or mood stabilizers with fewer side effects.

## References

[1] Mrazek F, Onderkova J, Szotkowski T (2014). Somatic mutation in acute myelogenous leukemia cells imitate novel germline HLA-A allele: a case report. Tissue Antigens.

[2] Dallaspezia S, Suzuki M, Benedetti F (2015). Chronobiological therapy for mood disorders. Curr Psychiatry Rep.

[3] van Calker D, Biber K, Domschke K (2019). The role of adenosine receptors in mood and anxiety disorders. J Neurochem.

[4] Gomes JI, Farinha-Ferreira M, Rei N (2021). Of adenosine and the blues: the adenosinergic system in the pathophysiology and treatment of major depressive disorder. Pharmacol Res.

[5] Szopa A, Socala K, Serefko A (2021). Purinergic transmission in depressive disorders. Pharmacol Ther.

[6] Reichert CF, Maire M, Schmidt C, Cajochen C (2016) Sleep-wake regulation and its impact on working memory performance: the role of adenosine. Biology (Basel) 5(1). 10.3390/biology5010011 10.3390/biology5010011 PMC4810168 26861410

[7] Lindberg D, Andres-Beck L, Jia YF (2018). Purinergic signaling in neuron-astrocyte interactions, circadian rhythms, and alcohol use disorder. Front Physiol.

[8] Wang XL LX, Yuan K, Han Y, Xue YY, Meng SQ, Li SX (2021) Clock genes Period1 and Period2 in the hippocampal CA1 mediate depression-like behaviors and rapid antidepressant response. BioRxiv. 10.1101/2021.08.14.456364

[9] Serchov T, Clement HW, Schwarz MK (2015). Increased signaling via adenosine A1 receptors, sleep deprivation, imipramine, and ketamine inhibit depressive-like behavior via induction of Homer1a. Neuron.

[10] Serchov T, Heumann R, van Calker D (2016). Signaling pathways regulating Homer1a expression: implications for antidepressant therapy. Biol Chem.

[11] Serchov T, Schwarz I, Theiss A (2020). Enhanced adenosine A1 receptor and Homer1a expression in hippocampus modulates the resilience to stress-induced depression-like behavior. Neuropharmacology.

[12] Holz A, Mulsch F, Schwarz MK (2019). Enhanced mGlu5 signaling in excitatory neurons promotes rapid antidepressant effects via AMPA receptor activation. Neuron.

[13] Sato S, Bunney BG, Vawter MP (2020). Homer1a undergoes bimodal transcriptional regulation by CREB and the circadian clock. Neuroscience.

[14] Pedata F, Dettori I, Coppi E (2016). Purinergic signalling in brain ischemia. Neuropharmacology.

[15] Moidunny S, Vinet J, Wesseling E (2012). Adenosine A2B receptor-mediated leukemia inhibitory factor release from astrocytes protects cortical neurons against excitotoxicity. J Neuroinflammation.

[16] Hines DJ, Schmitt LI, Hines RM (2013). Antidepressant effects of sleep deprivation require astrocyte-dependent adenosine mediated signaling. Transl Psychiatry.

[17] Serchov T, Atas HC, Normann C (2012). Genetically controlled upregulation of adenosine A(1) receptor expression enhances the survival of primary cortical neurons. Mol Neurobiol.

[18] Lewis KS, -Smith KG, Forty L,  (2017). Sleep loss as a trigger of mood episodes in bipolar disorder: individual differences based on diagnostic subtype and gender. Br J Psychiatry.

[19] Gubert C, Jacintho Moritz CE, Vasconcelos-Moreno MP (2016). Peripheral adenosine levels in euthymic patients with bipolar disorder. Psychiatry Res.

[20] Coelho JE, Alves P, Canas PM (2014). Overexpression of adenosine A2A receptors in rats: effects on depression, locomotion, and anxiety. Front Psychiatry.

[21] El Yacoubi M, Ledent C, Parmentier M (2001). Adenosine A2A receptor antagonists are potential antidepressants: evidence based on pharmacology and A2A receptor knockout mice. Br J Pharmacol.

[22] El Yacoubi M, Costentin J, Vaugeois JM (2003). Adenosine A2A receptors and depression. Neurology.

[23] Yamada K, Kobayashi M, Shiozaki S (2014). Antidepressant activity of the adenosine A2A receptor antagonist, istradefylline (KW-6002) on learned helplessness in rats. Psychopharmacology.

[24] Tsai SJ, Hong CJ, Hou SJ (2006). Association study of adenosine A2a receptor (1976C>T) genetic polymorphism and mood disorders and age of onset. Psychiatr Genet.

[25] Bartoli F, Clerici M, Carra G (2020). Purinergic system and suicidal behavior: exploring the link between adenosine A2A receptors and depressive/impulsive features. Mol Psychiatry.

[26] Lucas M, O’Reilly EJ, Pan A (2014). Coffee, caffeine, and risk of completed suicide: results from three prospective cohorts of American adults. World J Biol Psychiatry.

[27] Albrecht U (2012). Timing to perfection: the biology of central and peripheral circadian clocks. Neuron.

[28] Reppert SM, Weaver DR (2002). Coordination of circadian timing in mammals. Nature.

[29] Preitner N, Damiola F, Lopez-Molina L (2002). The orphan nuclear receptor REV-ERBalpha controls circadian transcription within the positive limb of the mammalian circadian oscillator. Cell.

[30] Sato TK, Panda S, Miraglia LJ (2004). A functional genomics strategy reveals Rora as a component of the mammalian circadian clock. Neuron.

[31] McClung CA (2013). How might circadian rhythms control mood? Let me count the ways. Biol Psychiatry.

[32] Ketchesin KD, Becker-Krail D, McClung CA (2020). Mood-related central and peripheral clocks. Eur J Neurosci.

[33] Wang XL, Wang DQ, Jiao FC (2021). Diurnal rhythm disruptions induced by chronic unpredictable stress relate to depression-like behaviors in rats. Pharmacol Biochem Behav.

[34] Mendoza J (2019). Circadian insights into the biology of depression: Symptoms, treatments and animal models. Behav Brain Res.

[35] Chellappa SL (2020). Circadian misalignment: a biological basis for mood vulnerability in shift work. Eur J Neurosci.

[36] Mendoza J, Vanotti G (2019). Circadian neurogenetics of mood disorders. Cell Tissue Res.

[37] Gutiérrez-Zotes A, Díaz-Peña R, Costas J (2020). Interaction between the functional SNP rs2070951 in NR3C2 gene and high levels of plasma corticotropin-releasing hormone associates to postpartum depression. Arch Womens Ment Health.

[38] Orozco-Solis R, Montellier E, Aguilar-Arnal L (2017). A circadian genomic signature common to ketamine and sleep deprivation in the anterior cingulate cortex. Biol Psychiatry.

[39] Duncan WC, Slonena E, Hejazi NS (2017). Motor-activity markers of circadian timekeeping are related to ketamine’s rapid antidepressant properties. Biol Psychiatry.

[40] Huhne A, Welsh DK, Landgraf D (2018). Prospects for circadian treatment of mood disorders. Ann Med.

[41] McClung CA (2007). Circadian genes, rhythms and the biology of mood disorders. Pharmacol Ther.

[42] McClung CA (2011). Circadian rhythms and mood regulation: insights from pre-clinical models. Eur Neuropsychopharmacol.

[43] Kohtala S, Alitalo O, Rosenholm M, Rozov S, Rantamaki T (2021) Time is of the essence: coupling sleep-wake and circadian neurobiology to the antidepressant effects of ketamine. Pharmacol Ther 221:107741. 10.1016/j.pharmthera.2020.107741 10.1016/j.pharmthera.2020.107741 33189715

[44] Wang XL, Yuan K, Zhang W (2020). Regulation of circadian genes by the MAPK pathway: implications for rapid antidepressant action. Neurosci Bull.

[45] Logan RW, McClung CA (2019). Rhythms of life: circadian disruption and brain disorders across the lifespan. Nat Rev Neurosci.

[46] Christiansen SL, Bouzinova EV, Fahrenkrug J et al (2016) Altered expression pattern of clock genes in a rat model of depression. Int J Neuropsychopharmacol 19.10.1093/ijnp/pyw061 10.1093/ijnp/pyw061 PMC5137278 27365111

[47] Li SX, Liu LJ, Xu LZ (2013). Diurnal alterations in circadian genes and peptides in major depressive disorder before and after escitalopram treatment. Psychoneuroendocrinology.

[48] Li JZ, Bunney BG, Meng F (2013). Circadian patterns of gene expression in the human brain and disruption in major depressive disorder. Proc Natl Acad Sci U S A.

[49] Etain B, Milhiet V, Bellivier F (2011). Genetics of circadian rhythms and mood spectrum disorders. Eur Neuropsychopharmacol.

[50] Lee KY, Song JY, Kim SH (2010). Association between CLOCK 3111T/C and preferred circadian phase in Korean patients with bipolar disorder. Prog Neuropsychopharmacol Biol Psychiatry.

[51] Soria V, Martinez-Amoros E, Escaramis G (2010). Differential association of circadian genes with mood disorders: CRY1 and NPAS2 are associated with unipolar major depression and CLOCK and VIP with bipolar disorder. Neuropsychopharmacology.

[52] Mansour HA, Talkowski ME, Wood J (2009). Association study of 21 circadian genes with bipolar I disorder, schizoaffective disorder, and schizophrenia. Bipolar Disord.

[53] Partonen T, Treutlein J, Alpman A (2007). Three circadian clock genes Per2, Arntl, and Npas2 contribute to winter depression. Ann Med.

[54] Landgraf D, McCarthy MJ, Welsh DK (2014). The role of the circadian clock in animal models of mood disorders. Behav Neurosci.

[55] Landgraf D, Long JE, Welsh DK (2016). Depression-like behaviour in mice is associated with disrupted circadian rhythms in nucleus accumbens and periaqueductal grey. Eur J Neurosci.

[56] Logan RW, Edgar N, Gillman AG (2015). Chronic stress induces brain region-specific alterations of molecular rhythms that correlate with depression-like behavior in mice. Biol Psychiat.

[57] Salaberry NL, Hamm H, Felder-Schmittbuhl MP (2019). A suprachiasmatic-independent circadian clock(s) in the habenula is affected by Per gene mutations and housing light conditions in mice. Brain Struct Funct.

[58] Meerlo P, van den Hoofdakker RH, Koolhaas JM (1997). Stress-induced changes in circadian rhythms of body temperature and activity in rats are not caused by pacemaker changes. J Biol Rhythms.

[59] Savalli G, Diao W, Schulz S, Todtova K, Pollak DD (2014) Diurnal oscillation of amygdala clock gene expression and loss of synchrony in a mouse model of depression. Int J Neuropsychopharmacol 18(5). 10.1093/ijnp/pyu095 10.1093/ijnp/pyu095 PMC4376549 25522426

[60] Sato S, Bunney B, Mendoza-Viveros L (2022). Rapid-acting antidepressants and the circadian clock. Neuropsychopharmacology.

[61] Coyle CM, Laws KR (2015). The use of ketamine as an antidepressant: a systematic review and meta-analysis. Hum Psychopharmacol-Clin Exp.

[62] Bellet MM, Vawter MP, Bunney BG (2011). Ketamine influences CLOCK:BMAL1 function leading to altered circadian gene expression. PLoS ONE.

[63] Bunney BG, Li JZ, Walsh DM (2015). Circadian dysregulation of clock genes: clues to rapid treatments in major depressive disorder. Mol Psychiatry.

[64] Bunney BG, Bunney WE (2012). Rapid-acting antidepressant strategies: mechanisms of action. Int J Neuropsychopharmacol.

[65] Wisor JP, Pasumarthi RK, Gerashchenko D (2008). Sleep deprivation effects on circadian clock gene expression in the cerebral cortex parallel electroencephalographic differences among mouse strains (vol 28, pg 7193, 2008). J Neurosci.

[66] Trautmann C, Burek D, Hubner CA (2020). A regulatory pathway linking caffeine action, mood and the diurnal clock. Neuropharmacology.

[67] Robillard R, Hermens DF, Lee RS (2016). Sleep-wake profiles predict longitudinal changes in manic symptoms and memory in young people with mood disorders. J Sleep Res.

[68] Fang L, Yu Q, Yin F, Yu J, Zhang Y, Zhang Y, et al (2021) Combined cortisol and melatonin measurements with detailed parameter analysis can assess the circadian rhythms in bipolar disorder patients. Brain Behav 11(7):e02186. 10.1002/brb3.2186 10.1002/brb3.2186 PMC8323050 34096190

[69] McCarty R, Josephs T, Kovtun O (2021). Enlightened: addressing circadian and seasonal changes in photoperiod in animal models of bipolar disorder. Transl Psychiatry.

[70] Gonzalez R, Tohen M (2018) Circadian rhythm and the prediction of relapse in bipolar disorder. J Clin Psychiatry 79(1). 10.4088/JCP.17com11821 10.4088/JCP.17com11821 29286594

[71] Wehr TA (2018). Bipolar mood cycles associated with lunar entrainment of a circadian rhythm. Transl Psychiatry.

[72] Rosenthal SJ, Josephs T, Kovtun O (2020). Seasonal effects on bipolar disorder: a closer look. Neurosci Biobehav Rev.

[73] Gonzalez R (2014). The relationship between bipolar disorder and biological rhythms. J Clin Psychiatry.

[74] Rosenthal SJ, McCarty R (2019). Switching winter and summer photoperiods in an animal model of bipolar disorder. Neuropsychopharmacology.

[75] Porcu A, Gonzalez R, McCarthy MJ (2019). Pharmacological manipulation of the circadian clock: a possible approach to the management of bipolar disorder. CNS Drugs.

[76] Novakova M, Prasko J, Latalova K (2015). The circadian system of patients with bipolar disorder differs in episodes of mania and depression. Bipolar Disord.

[77] Moon JH, Cho CH, Son GH (2016). Advanced circadian phase in mania and delayed circadian phase in mixed mania and depression returned to normal after treatment of bipolar disorder. EBioMedicine.

[78] Inder ML, Crowe MT, Porter R (2016). Effect of transmeridian travel and jetlag on mood disorders: evidence and implications. Aust N Z J Psychiatry.

[79] Duarte Faria A, Cardoso Tde A, Campos Mondin T (2015). Biological rhythms in bipolar and depressive disorders: a community study with drug-naive young adults. J Affect Disord.

[80] Kristensen M, Nierenberg AA, Ostergaard SD (2018). Face and predictive validity of the ClockDelta19 mouse as an animal model for bipolar disorder: a systematic review. Mol Psychiatry.

[81] Logan RW, McClung CA (2016). Animal models of bipolar mania: the past, present and future. Neuroscience.

[82] Hampp G, Ripperger JA, Houben T (2008). Regulation of monoamine oxidase A by circadian-clock components implies clock influence on mood. Curr Biol.

[83] Olejniczak I, Ripperger JA, Sandrelli F (2021). Light affects behavioral despair involving the clock gene Period 1. PLoS Genet.

[84] Baldessarini RJ, Tondo L, Vazquez GH (2019). Pharmacological treatment of adult bipolar disorder. Mol Psychiatry.

[85] Hinton DJ, Andres-Beck LG, Nett KE (2019). Chronic caffeine exposure in adolescence promotes diurnal, biphasic mood-cycling and enhanced motivation for reward in adult mice. Behav Brain Res.

[86] Lazzerini Ospri L, Prusky G, Hattar S (2017). Mood, the circadian system, and melanopsin retinal ganglion cells. Annu Rev Neurosci.

[87] Antle MC, Steen NM, Mistlberger RE (2001). Adenosine and caffeine modulate circadian rhythms in the Syrian hamster. NeuroReport.

[88] Elliott KJ, Todd Weber E, Rea MA (2001). Adenosine A1 receptors regulate the response of the hamster circadian clock to light. Eur J Pharmacol.

[89] Sigworth LA, Rea MA (2003). Adenosine A1 receptors regulate the response of the mouse circadian clock to light. Brain Res.

[90] Porkka-Heiskanen T, Strecker RE, McCarley RW (2000). Brain site-specificity of extracellular adenosine concentration changes during sleep deprivation and spontaneous sleep: an in vivo microdialysis study. Neuroscience.

[91] Burgess HJ (2010). Partial sleep deprivation reduces phase advances to light in humans. J Biol Rhythms.

[92] van Diepen HC, Lucassen EA, Yasenkov R (2014). Caffeine increases light responsiveness of the mouse circadian pacemaker. Eur J Neurosci.

[93] Wisor JP, Pasumarthi RK, Gerashchenko D (2008). Sleep deprivation effects on circadian clock gene expression in the cerebral cortex parallel electroencephalographic differences among mouse strains. J Neurosci.

[94] Zhang B, Gao Y, Li Y (2016). Sleep Deprivation influences circadian gene expression in the lateral habenula. Behav Neurol.

[95] Maret S, Dorsaz S, Gurcel L (2007). Homer1a is a core brain molecular correlate of sleep loss. Proc Natl Acad Sci U S A.

[96] Thompson CL, Wisor JP, Lee CK (2010). Molecular and anatomical signatures of sleep deprivation in the mouse brain. Front Neurosci.

[97] Oike H, Kobori M, Suzuki T (2011). Caffeine lengthens circadian rhythms in mice. Biochem Biophys Res Commun.

[98] Jha PK, Bouaouda H, Gourmelen S (2017). Sleep deprivation and caffeine treatment potentiate photic resetting of the master circadian clock in a diurnal rodent. J Neurosci.

[99] Ruby CL, Verbanes NM, Palmer KN (2018). Caffeine delays light-entrained activity and potentiates circadian photic phase-resetting in mice. J Biol Rhythms.

[100] Burke TM, Markwald RR, McHill AW (2015). Effects of caffeine on the human circadian clock in vivo and in vitro. Sci Transl Med.

[101] Blendy JA (2006). The role of CREB in depression and antidepressant treatment. Biol Psychiatry.

[102] Maurer C, Winter T, Chen S (2016). The CREB-binding protein affects the circadian regulation of behaviour. FEBS Lett.

[103] Travnickova-Bendova Z, Cermakian N, Reppert SM (2002). Bimodal regulation of mPeriod promoters by CREB-dependent signaling and CLOCK/BMAL1 activity. Proc Natl Acad Sci U S A.

[104] Jajoo S, Mukherjea D, Kumar S (2010). Role of beta-arrestin1/ERK MAP kinase pathway in regulating adenosine A1 receptor desensitization and recovery. Am J Physiol Cell Physiol.

[105] O’Neill JS, Maywood ES, Chesham JE (2008). cAMP-dependent signaling as a core component of the mammalian circadian pacemaker. Science.

[106] Ikegami K, Nakajima M, Minami Y (2020). cAMP response element induces Per1 in vivo. Biochem Biophys Res Commun.

[107] Logan RW, Parekh PK, Kaplan GN (2019). NAD+ cellular redox and SIRT1 regulate the diurnal rhythms of tyrosine hydroxylase and conditioned cocaine reward. Mol Psychiatry.

[108] Xiang L, Feng Y, Hu Q (2020). Jiao-Tai-Wan ameliorates depressive-like behavior through the A1R pathway in ovariectomized mice after unpredictable chronic stress. Biomed Res Int.

[109] Qi H, Mailliet F, Spedding M (2009). Antidepressants reverse the attenuation of the neurotrophic MEK/MAPK cascade in frontal cortex by elevated platform stress; reversal of effects on LTP is associated with GluA1 phosphorylation. Neuropharmacology.

[110] Duric V, Banasr M, Licznerski P (2010). A negative regulator of MAP kinase causes depressive behavior. Nat Med.

[111] Zhang L, Xu T, Wang S (2012). Curcumin produces antidepressant effects via activating MAPK/ERK-dependent brain-derived neurotrophic factor expression in the amygdala of mice. Behav Brain Res.

[112] Di Benedetto B, Radecke J, Schmidt MV (2013). Acute antidepressant treatment differently modulates ERK/MAPK activation in neurons and astrocytes of the adult mouse prefrontal cortex. Neuroscience.

[113] Wang JQ, Mao L (2019). The ERK pathway: molecular mechanisms and treatment of depression. Mol Neurobiol.

[114] Yuan S, Jiang X, Zhou X (2018). Inosine alleviates depression-like behavior and increases the activity of the ERK-CREB signaling in adolescent male rats. NeuroReport.

[115] O’Neill JS, Reddy AB (2012). The essential role of cAMP/Ca2+ signalling in mammalian circadian timekeeping. Biochem Soc Trans.

[116] Wang XL, Gao J, Wang XY (2018). Treatment with Shuyu capsule increases 5-HT1AR level and activation of cAMP-PKA-CREB pathway in hippocampal neurons treated with serum from a rat model of depression. Mol Med Rep.

[117] Yamashiro K, Fujii Y, Maekawa S (2017). Multiple pathways for elevating extracellular adenosine in the rat hippocampal CA1 region characterized by adenosine sensor cells. J Neurochem.

[118] Hastings MH, Maywood ES, O’Neill JS (2008). Cellular circadian pacemaking and the role of cytosolic rhythms. Curr Biol.

[119] Brancaccio M, Maywood ES, Chesham JE (2013). A Gq-Ca2+ axis controls circuit-level encoding of circadian time in the suprachiasmatic nucleus. Neuron.

[120] Aguilar-Roblero R, Mercado C, Alamilla J (2007). Ryanodine receptor Ca2+-release channels are an output pathway for the circadian clock in the rat suprachiasmatic nuclei. Eur J Neurosci.

[121] Yuan P, Zhou R, Wang Y (2010). Altered levels of extracellular signal-regulated kinase signaling proteins in postmortem frontal cortex of individuals with mood disorders and schizophrenia. J Affect Disord.

[122] Jagannath A, Varga N, Dallmann R (2021). Adenosine integrates light and sleep signalling for the regulation of circadian timing in mice. Nat Commun.

[123] Johansson B, Georgiev V, Fredholm BB (1997). Distribution and postnatal ontogeny of adenosine A2A receptors in rat brain: comparison with dopamine receptors. Neuroscience.

[124] Fuxe K, Ferre S, Genedani S (2007). Adenosine receptor-dopamine receptor interactions in the basal ganglia and their relevance for brain function. Physiol Behav.

[125] Ferre S (2008). An update on the mechanisms of the psychostimulant effects of caffeine. J Neurochem.

[126] Radwan B, Liu H, Chaudhury D (2019). The role of dopamine in mood disorders and the associated changes in circadian rhythms and sleep-wake cycle. Brain Res.

[127] Chen AC, Shirayama Y, Shin KH (2001). Expression of the cAMP response element binding protein (CREB) in hippocampus produces an antidepressant effect. Biol Psychiatry.

[128] Pliakas AM, Carlson RR, Neve RL (2001). Altered responsiveness to cocaine and increased immobility in the forced swim test associated with elevated cAMP response element-binding protein expression in nucleus accumbens. J Neurosci.

[129] Wallace TL, Stellitano KE, Neve RL (2004). Effects of cyclic adenosine monophosphate response element binding protein overexpression in the basolateral amygdala on behavioral models of depression and anxiety. Biol Psychiatry.

[130] Lamph WW, Dwarki VJ, Ofir R (1990). Negative and positive regulation by transcription factor camp response element-binding protein is modulated by phosphorylation. Proc Natl Acad Sci USA.

[131] Masquilier D, Sassone-Corsi P (1992). Transcriptional cross-talk: nuclear factors CREM and CREB bind to AP-1 sites and inhibit activation by Jun. J Biol Chem.

[132] Fiore P, Gannon RL (2003). Expression of the transcriptional coactivators CBP and p300 in the hamster suprachiasmatic nucleus: possible molecular components of the mammalian circadian clock. Brain Res Mol Brain Res.

[133] van Calker D, Serchov T, Normann C (2018). Recent insights into antidepressant therapy: distinct pathways and potential common mechanisms in the treatment of depressive syndromes. Neurosci Biobehav Rev.

[134] Dallaspezia S, Benedetti F (2015). Sleep deprivation therapy for depression. Curr Top Behav Neurosci.

[135] Elmenhorst D, Basheer R, McCarley RW (2009). Sleep deprivation increases A(1) adenosine receptor density in the rat brain. Brain Res.

[136] Kim Y, Elmenhorst D, Weisshaupt A (2015). Chronic sleep restriction induces long-lasting changes in adenosine and noradrenaline receptor density in the rat brain. J Sleep Res.

[137] Dias RB, Rombo DM, Ribeiro JA (2013). Adenosine: setting the stage for plasticity. Trends Neurosci.

[138] Diering GH, Nirujogi RS, Roth RH (2017). Homer1a drives homeostatic scaling-down of excitatory synapses during sleep. Science.

[139] Kaster MP, Rosa AO, Rosso MM (2004). Adenosine administration produces an antidepressant-like effect in mice: evidence for the involvement of A1 and A2A receptors. Neurosci Lett.

[140] Yamada K, Kobayashi M, Kanda T (2014). Involvement of adenosine A2A receptors in depression and anxiety. Int Rev Neurobiol.

